# Sociodemographic disparities in survival from colorectal cancer in South Australia: a population-wide data linkage study

**DOI:** 10.1186/s12913-016-1263-3

**Published:** 2016-01-20

**Authors:** Kerri R. Beckmann, Alice Bennett, Graeme P. Young, Stephen R. Cole, Rohit Joshi, Jacqui Adams, Nimit Singhal, Christos Karapetis, David Wattchow, David Roder

**Affiliations:** 1 Centre for Population Health Research, University of South Australia, GPO Box 2471, Adelaide, SA 5001 Australia; 2 Flinders Medical Centre, Flinders Drive, Bedford Park, SA 5042 Australia; 3 Flinders Centre for Innovation in Cancer, Flinders University, Flinders Drive, Bedford Park, SA 5042 Australia; 4 Country Health SA, Adelaide, SA 5000 Australia; 5 Lyell McEwin Hospital, Elizabeth Vale, SA 5112 Australia; 6 Medical Oncologist, Royal Adelaide Hospital, University of Adelaide, Adelaide, SA 5001 Australia; 7 South Adelaide Health Network, Medical Oncology, Flinders Medical Centre, Flinders Drive, Bedford Park, SA 5042 Australia; 8 Flinders University, Flinders Medical Centre, Bedford Park, SA 5042 Australia

**Keywords:** Colorectal cancer, Socio-demographic inequalities, Stage, Survival

## Abstract

**Background:**

Inequalities in survival from colorectal cancer (CRC) across socioeconomic groups and by area of residence have been described in various health care settings. Few population-wide datasets which include clinical and treatment information are available in Australia to investigate disparities. This study examines socio-demographic differences in survival for CRC patients in South Australia (SA), using a population-wide database derived via linkage of administrative and surveillance datasets*.*

**Methods:**

The study population comprised all cases of CRC diagnosed in 2003-2008 among SA residents aged 50-79 yrs in the SA Central Cancer Registry. Measures of socioeconomic status (area level), geographical remoteness, clinical characteristics, comorbid conditions, treatments and outcomes were derived through record linkage of central cancer registry, hospital-based clinical registries, hospital separations, and radiotherapy services data sources. Socio-demographic disparities in CRC survival were examined using competing risk regression analysis.

**Results:**

Four thousand six hundred and forty one eligible cases were followed for an average of 4.7 yrs, during which time 1525 died from CRC and 416 died from other causes. Results of competing risk regression indicated higher risk of CRC death with higher grade (HR high v low =2.25, 95 % CI 1.32-3.84), later stage (HR C v A = 7.74, 95 % CI 5.75-10.4), severe comorbidity (HR severe v none =1.21, 95 % CI 1.02-1.44) and receiving radiotherapy (HR = 1.41, 95 % CI 1.18-1.68). Patients from the most socioeconomically advantaged areas had significantly better outcomes than those from the least advantaged areas (HR =0.75, 95 % 0.62-0.91). Patients residing in remote locations had significantly worse outcomes than metropolitan residents, though this was only evident for stages A-C (HR = 1.35, 95 % CI 1.01-1.80). These disparities were not explained by differences in stage at diagnosis between socioeconomic groups or area of residence. Nor were they explained by differences in patient factors, other tumour characteristics, comorbidity, or treatment modalities.

**Conclusions:**

Socio-economic and regional disparities in survival following CRC are evident in SA, despite having a universal health care system. Of particular concern is the poorer survival for patients from remote areas with potentially curable CRC. Reasons for these disparities require further exploration to identify factors that can be addressed to improve outcomes.

## Background

Rates of colorectal cancer (CRC) in Australia are among the highest in the world [1]. CRC is the second most commonly reported cancer in Australia, after prostate cancer and second most common cause of cancer death, after lung cancer, with approximately 16,000 new cases and 4000 deaths in 2012 [2]. The cost of managing and treating CRC exceeds that for any other cancer, surpassing A$427 million for 2008/09 [3]. While survival from CRC is relatively favourable, outcomes are highly dependent on stage at diagnosis. Currently in Australia, five year relative survival is 86 % for localised CRC compared with 66 % for regional disease and only 12 % for distant spread [4]. However, only one third of CRCs are ‘localised’ at diagnosis [5]. Earlier detection of CRC should therefore lead to substantial improvements in survival [6].

Reducing inequalities is an increasingly important focus of cancer control efforts, alongside improving survival overall. Socioeconomic and regional inequalities in survival from CRC have been observed internationally [7–16], and in Australia [17–19], despite many countries having universal healthcare. Reasons for sociodemographic differences in outcomes are not clear. Lower socioeconomic status (SES) is generally associated with later stage at diagnosis, and in some settings, poorer standards of care, less favourable health behaviours, and, or greater co-morbidity [20, 21]. Geographic variation may be due to lack of access to cancer screening and diagnostic services leading to later stage at diagnosis. Increased distance to cancer treatment services may deter or restrict patients from accessing or completing treatment, leading to disparities in treatment with consequent impacts on survival among rural patients [22–24]. Additionally limited follow-up facilities in remote locations may impact survival.

National data for Australia indicate disparities in CRC survival according to remoteness of residence and socioeconomic status at the area level [3]. Five-year relative survival for remote residents was 62.8 % compared with 67.2 % for those living in major cities, and 64.5 % for those residing in the lowest SES quintile compared with 69.4 % in the highest SES quintile. Whether these disparities reflect differences in stage at diagnosis is unclear, due to the lack staging information in Australian cancer registries. Nor have any national or state-wide studies been undertaken covering both the public and private sector that include stage, comorbidity and treatment data to disentangle the impact of these factors on socioeconomic disparities.

This study aimed to identify factors associated with disparities in CRC survival using population-wide data from South Australia. Specifically we examined whether CRC survival differs according to area level measures of socioeconomic disadvantage and remoteness of residence. We also examined factors associated with stage at diagnosis, which may in part explain any survival differences we observe. We hypothesis that we should not see any disparities in survival according to area-level measures of disadvantage, after adjustment for stage, treatment and comorbidities, since healthcare is freely available to all Australian residents. On the other hand we would expect survival to vary according to place of residence, with poorer outcomes for more isolated residents due to limited access to healthcare services.

In Australia, healthcare services are generally delivered at the State level, along with administrative and surveillance data systems accompanying service delivery. Thus, population-based health services research is most feasible at the state level. While the population of South Australia is relatively small (1.5 million, approximately 8 % of Australia’s total population), the geographic and socioeconomic variation across the state makes it an ideal population in which to explore disparities in CRC survival. Given the commonality in healthcare models across states, and the similarity with the nation-wide population profile, findings from South Australia will give insight into the nature of disparities at the national level. Further, they may confirm international findings suggesting socioeconomic and regional disparities in CRC survival despite universal healthcare.

## Methods

### Data sources and linkage

The study population comprised SA residents diagnosed with CRC (ICD10 C18-20) from January 2003 to December 2008, aged 50 and 79 years, identified from the South Australia Cancer Registry (SACR). This age range and diagnostic period corresponds to cases that were staged for a previous study on bowel cancer screening. Cases of squamous cell carcinoma, melanoma, carcinoid and baseloid tumours, perianal Paget’s disease, gastrointestinal stromal tumours and cancers of unknown primary origin were excluded, since they are generally staged and managed quite differently. Synchronous colon and rectal cancer cases (*n* = 47) were classified together with rectal cancers.

**Fig. 1:**
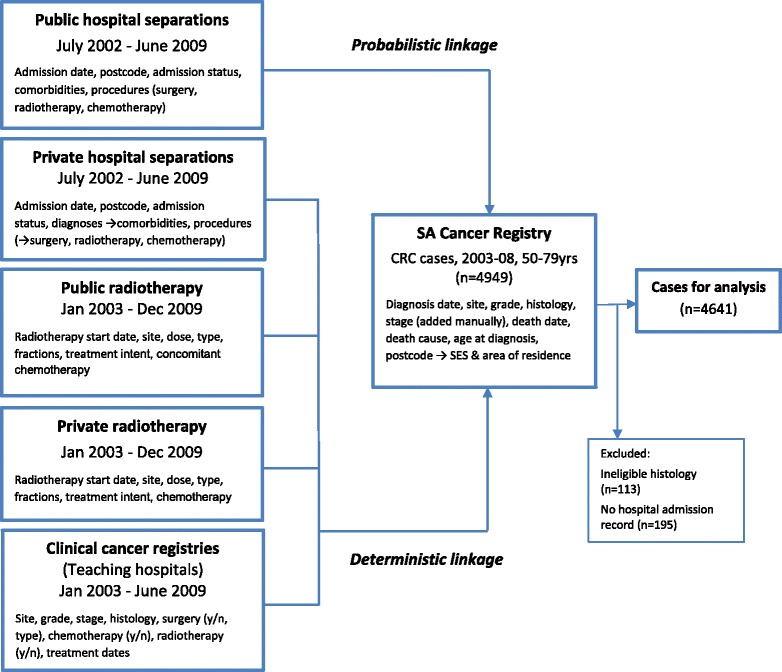
Data sources and linkages used to compile study dataset

A mixture of probabilistic and deterministic linkage methods were used to develop a comprehensive population-wide dataset incorporating demographic, clinical and outcome data for all CRC cases. SACR records pertaining to eligible cases constituted the core dataset. Linkage between the SACR and public hospital separation data was undertaken by SANT DataLink using probabilistic matching based on name, address and date of birth, to retrieve records pertaining to all admissions for each individual, which included an admission code for CRC. Linkage between the SACR and private hospital separation data, radiotherapy services and hospital-based cancer registries was undertaken by the Department of Health through direct matching of hospital codes, patient record numbers, cancer registry accession number and personal identifiers, again to identify all relevant records pertaining to CRC diagnosis or treatment from these data sources. SANT DataLink linkage processes followed the best practice principle of ‘data separation’ to ensure privacy and anonymity [25]. Figure 1 shows the data sources and linkage processes used in the construction of this dataset.

### Measures

All eligible cases had previously been manually staged by SACR staff for a previous study [5], according to Australian Clinico-Pathological Staging (ACPS) criteria [26], based on data from pathology reports, supplemented by electronic patient records and hospital-based cancer registry (HBCR) data. Grade of the primary tumour at diagnosis was extracted from SACR records and was coded as low, intermediate or high grade corresponding to well-differentiated, moderately-differentiated and poorly-differentiated tumours based on pathological findings. Stage and grade were unknown in 278 (6.0 %) and 362 cases (7.8 %), respectively.

Area of residence was classified into 5 categories:- inner urban (very accessible), outer urban (moderately accessible), rural (accessible), remote and very remote, according to the Accessibility/Remoteness Index of Australia, 2006 [27], based on residential postcode at diagnosis as recorded by the SACR. This measure describes place of residence according to road distance from population service centres. Remote and very remote categories were combined for analyses due to small numbers. Similarly, an area level measure of SES was assigned using the Index of Relative Socioeconomic Advantage and Disadvantage (IRSAD) 2006 [28], based on residential postcode. This index measures area-level rather than individual-level characteristics and represents the average socioeconomic status of specified neighbourhoods (in this case postal areas). It incorporates factors such as average household income, education levels and unemployment rates. SES was categorised into quintiles corresponding to SA's population distribution. No individual level measures of SES were available. Private health insurance status was assigned if any individuals’ hospital admissions flagged the patient as being privately insured. Patient co-morbidity was assessed using the Charlson co-morbidity index (CCI) [29] for the patient’s first admission for surgical treatment or their first admission for CRC if there was no surgical admission. Diagnosis codes for CRC and metastatic disease were excluded when calculating the CCI. CCI scores were further categorised into no co-morbidity (CCI = 0), a single co-morbidity (CCI =1) or multiple co-morbidities (CCI > =2).

Treatments were assessed from multiple sources and coded dichotomously (yes/no) based on any indication in any data source (i.e. any procedure codes for relevant treatment in hospital admissions, HBCR or radiotherapy records). ‘Primary treatment’ was defined as treatment commencing within 12 months of diagnosis, based on the date of the first hospital admission or the treatment start date in the HBCR or radiotherapy datasets if there was no hospital admission record for that treatment.

Information about vital status was derived from the SACR, which routinely links with state and national death notifications to track dates and causes of death occurring in any Australian jurisdiction.

### Analysis

Ordinal logistic regression was undertaken to identify factors independently associated with stage at diagnosis. ACPS stage, categorised as four separate stages (A-D), was the dependent variable. Unknown stage was excluded. Adjusted odds ratios were obtained from a multivariate model adjusting for all covariates simultaneously (age group, sex, cancer site, SES (quintiles), place of residence, co-morbidity, primary surgery, radiotherapy, and chemotherapy, and calendar year of diagnosis (continuous). Cancer-specific survival was examined crudely, in the first instance, using Kaplan-Meier product limit estimates without taking competing risk into account, with differences assessed using log rank tests. We then assessed factors independently associated with risk of CRC death, using competing risk regression analysis according to the method of Fine and Gray [30], where death from other causes was the competing risk. Cases were censored on December 31, 2012 or at their date of death (whichever occurred earlier). Follow-up time was calculated as the time from the date of diagnosis to the censoring date or date of death if death occurred before this date. Multivariable models included cancer site, stage, grade, age, sex, year of diagnosis, comorbidity categories, SES, place of residence, and primary treatments received. Cases with missing stage were excluded from analyses, whereas cases with missing grade were included as a separate ‘unknown’ category. We repeated the competing risk analysis with imputed data for missing stage and grade, derived using multiply imputed chained equations [31], with 20 imputed data sets. Only complete case analyses are reported since results did not differ substantially for multiply imputed data. All analyses were undertaken using Stata v12.0 software [32].

This study received ethical approval from Human Research Ethics Committees of SA Health, the Royal Adelaide Hospital, Flinders University/Medical Centre and the SA Aboriginal Health Council, along with data access approval from SA Health for government managed datasets.

## Results

### Dataset profile

Of the 4949 CRC cases identified from the SACR, 113 were excluded due to ineligible histology, and 195 due to lack of hospital admission records, leaving a total of 4641 for analysis. The average period of follow-up was 4.7 yrs, corresponding to 6.3 yrs among survivors and 2.3 yrs among those who died of CRC or other causes. Demographic and clinical characters of the study cohort are presented in Table 1.

**Table 1:** Characteristics of the study population (CRC diagnosed among SA residents 50-79 yrs, 2003-2008)

Factor		Total no. (%)
Total	Number (% of total)	4641 (100)
Sex	Female	1973 (42.5)
	Male	2668 (57.5)
Age	50-59 yrs	959 (20.7)
	60-69 yrs	1601 (34.5)
	70-79 yrs	2085 (44.8)
Place of	Inner urban	3304 (71.2)
residence	Outer urban	459 (10.0)
	Rural	621 (13.4)
	Remote	257 (5.5)
Socioeconomic	Lowest quintile	1120 (24.1)
status	Mid-low	939 (20.2)
	Mid	849 (18.3)
	Mid-high	897 (19.3)
	Highest quintile	836 (18.0)
Private Insurance	No	2082 (44.9)
	Yes	2559 (55.1)
Year	2003	789 (17.0)
	2004	771 (16.6)
	2005	722 (15.6)
	2006	744 (16.0)
	2007	828 (17.8)
	2008	787 (17.0)
Stage#	A	933 (20.4)
	B	1438 (30.1)
	C	1345 (28.0)
	D	649 (14.0)
	*unknown*	278 (6.0)
Grade	Low	123 (2.7)
	Intermediate	3293 (71.0)
	High	863 (18.6)
	*unknown*	362 (7.8)
Comorbidity^*^	None	3236 (69.7)
	Single comorbidity	819 (17.7)
	Severe or multiple	586 (12.6)
Site	Colon	3005 (64.7)
	Rectal or both	1636 (35.3)

*Comorbidity assessed using Charlson comorbidity index

#ACPS -Australian clinico-pathological staging

### Stage at diagnosis

In unadjusted analyses, distribution of stage at diagnosis did not differ by remoteness of residence or SES, but did differ by cancer site, age group, extent of comorbidity and health insurance status (Table 2). Multivariate analysis using ordinal logistic regression indicates that later stage was less likely among rectal cancer cases (0R = 0.81, 95 % CI 0.72-0.91), older patients (0R = 0.77, 95 % CI 0.67-0.89 for 70-79 yrs v 50-59 yrs) and privately insured patients (OR = 0.84, 95 % CI 0.75-0.95) and more likely for those with co-morbidities (OR = 1.50, 95 % CI 1.26-1.79 for CCI > = 2 v 0). Stage at diagnosis was not associated with socioeconomic status (area-level), place of residence, or sex in multivariate analyses.

**Table 2:** Crude and adjusted odds ratios for factors associated with later stage at diagnosis derived from ordinal logistic regression (unknown stage excluded) *N* = 4362

			Crude			Adjusted#	
Variables		OR	95 % CI	*p*-value	OR	95 % CI	*p*-value
Site	Colon	1.00			1.00		
	Rectum	0.82	0.73-0.91	<0.001	0.81	0.72-0.91	<0.001
Age group	50-59 yrs	1.00			1.00		
	60-69 yrs	0.82	0.70-0.95	<0.008	0.76	0.66-0.89	<0.001
	70-79 yrs	0.88	0.77-1.02	0.087	0.77	0.67-0.89	0.001
Sex	Females	1.00			1.00		
	Males	0.97	0.87-1.08	0.562	0.96	0.86-1.07	0.436
SES	Lowest (quintile)	1.00			1.00		
	Low	0.89	0.75-1.06	0.208	0.90	0.75-1.08	0.248
	Mid	0.99	0.83-1.18	0.909	0.96	0.79-1.15	0.636
	Low-high	0.93	0.78-1.10	0.395	0.90	0.75-1.08	0.268
	Highest	1.08	0.91-1.26	0.438	1.03	0.86-1.23	0.756
Residence	Inner urban	1.00			1.00		
	Outer urban	0.85	0.70-1.05	0.080	0.89	0.74-1.07	0.248
	Rural	1.02	0.88-1.20	0.717	0.99	0.83-1.16	0.812
	Remote	0.92	0.73-1.16	0.473	0.99	0.77-1.26	0.912
Private Insurance	No	1.00			1.00		
	Yes	0.88	0.76-0.94	0.002	0.84	0.75-0.95	0.003
Co-morbidities	None	1.00			1.00		
	One (not severe)	1.25	1.09-1.44	0.002	1.25	1.09-1.44	0.002
	Multiple or severe	1.48	1.25-1.76	<0.001	1.50	1.26-1.79	<0.001
Diagnosis year	2003-2008 (cont)	0.97	0.94-1.00	0.024	0.97	0.94-1.00	0.027

#Ordinal logistic regression model adjusted for all factors simultaneously – excludes missing stage (*n* = 328)

Ordinal logistic regression is an extension of logistic regression that incorporates the ordinal nature of the dependent variable (in this case stage at diagnosis). It can be used for modelling a dependant variable that has more than two ordered categories. The method is analogous to a series of binary models predicting the following combinations of binary stage groupings (e.g. stage A v stage B + C + D, A + B v C + D and A + B + C v D)

### Survival outcomes

CRC specific survival was 88 %, 75 % and 69 % at 1, 3 and 5 years respectively (Table 3). In crude analysis, survival was strongly associated with stage of disease, grade of tumour, age at diagnosis and severity of co-morbid conditions. Survival was also associated with receipt of specific treatments (with better survival among those having surgery compared with no surgery and worse survival for those who had radiotherapy or chemotherapy compared with those not receiving these treatments. There were no significant differences by place of residence but survival did differ across SES quintiles and according to whether patients were privately insured. Multivariate competing risk regression (Table 4) revealed similar results, though there were some slight differences to crude survival analyses. Risk of CRC death was higher among patients with later stage disease (e.g. HR = 7.74, 95 % CI 5.75-10.4 for stage C vs stage A), higher grade (HR = 2.25, 95 % CI 1.32-3.84 for high verses low grade), and severe or multiple co-morbidities compared with no comorbidity (HR = 1.22, 95 % CI 1.02-1.44) and lower among rectal compared with colon cancer patients (HR = 0.85, 95 % CI 0.72-0.98). However there were no significant differences by age group or by health insurance status after adjusting for other covariates and accounting for death from other causes. Risk of CRC death was significantly lower among those who underwent surgery (HR = 0.51 95 % CI 0.42-0.62) and higher among those who received radiotherapy (HR = 1.41, 95 % CI 1.18-1.68), but unlike crude analyses, CRC death was lower among those who received chemotherapy (HR = 0.87 95 % CI 0.76-1.00). No differences were found according to place of residence, however risk was significantly lower among the highest compared with lowest SES group (HR = 0.75, 95 % CI 0.62-0.91). Risk of CRC death declined significantly over the study period (HR = 0.95, 95 % CI 0.92-0.98).

**Table 3:** 1, 3 and 5 year colorectal cancer cancer-specific survival [%], for South Australian residents aged 50-79 yr, diagnosed 2003-2008 (unadjusted). [*N* = 8461]

	CRC-survival (%)	1 yr	95 % CI	3 yrs	95 % CI	5 yrs	95 % CI	*p*-value#
Total		88	87 - 89	75	73 - 76	69	68 - 71	
Age group	50-59 yrs	91	89 - 92	75	72 - 78	68	65 - 71	
	60-69 yrs	90	88 - 91	76	74 - 79	70	67 - 72	0.016
	70-79 yrs	85	84 - 87	71	69 - 72	66	64 - 68	
Sex	Female	88	86 - 89	74	72 - 76	69	66 - 71	0.156
	Male	88	87 - 89	74	72 - 75	67	65 - 69	
Private Insurance	No	85	83 - 86	71	69 - 73	65	62 - 67	<0.001
	Yes	91	89 - 92	76	75 - 79	70	68 - 72	
Residence	Inner urban	88	87 - 89	75	72 - 76	69	66 - 69	
	Outer urban	87	84 - 90	76	71 - 80	71	66 - 75	0.219
	Rural	86	83 - 89	74	69 - 76	68	64 - 71	
	Remote	89	83 - 92	73	66 - 79	64	56 - 70	
	Very remote	84	67 - 92	75	57 - 86	59	38 - 76	
SES (quintiles)	Least advantaged	86	84 - 88	70	69 - 75	64	61 - 67	
	Mid-low	87	85 - 89	74	71 - 77	68	65 - 71	
	Mid	87	85 - 89	74	72 - 78	69	66 - 72	0.010
	Mid-high	89	87 - 91	74	72 - 78	67	64 - 70	
	Most advantaged	91	89 - 93	78	75 - 81	71	68 - 74	
ACP Stage	A	98	97 - 99	97	95- 98	95	93 - 96	
	B	97	95 - 97	89	87 - 90	84	82 - 86	
	C	91	89 - 92	72	69 - 74	62	59 - 64	<0.001
	D	54	50 - 58	18	15 - 21	9	7 - 12	
	Unknown	72	66 - 77	58	51 - 63	56	49 - 62	
Grade	Low	93	87 - 97	87	79 - 92	85	77 - 90	
	Intermediate	93	92 - 94	81	80 - 82	75	73 - 76	<0.001
	High	77	74 - 79	55	52 - 58	49	46 - 53	
	Unknown	66	61 - 71	47	41 - 52	43	37 - 48	
Site	Colon	87	86 - 88	73	72 - 74	68	66 - 69	
	Rectum	90	88 - 91	76	73 - 78	68	66 - 70	0.747
Co-morbidity	None	90	89 - 91	77	75 - 78	70	69 - 72	
	One (not severe)	87	85 - 89	73	70 - 76	68	64 - 71	<0.001
	Multiple / severe	75	71 - 78	58	54 - 62	52	48 - 56	
Surgery†	No	46	41 - 51	24	20 - 28	19	16 - 24	<0.001
	Yes	92	91 - 93	79	77 - 80	73	71 - 74	
Radiotherapy†	No	88	87 - 89	75	74 - 76	70	68 - 71	<0.001
	Yes	87	84 - 89	67	63 - 71	57	53 - 61	
Chemotherapy†	No	88	87 - 89	81	79 - 82	77	75- 79	<0.001
	Yes	88	86 - 89	62	59 - 64	52	50 - 55	

#Kaplan-meier log rank test

†Treatments received within 12 months of diagnosis

Mean follow-up time to death or censoring of 40 months (sd = 24 months), median 36 months (inter-quartile range 21-59 months

**Table 4:** Multivariate competing risks regression analysis for risk of CRC death, all CRC patients

		CRC death/ no. at risk	All CRC (n = 4365)		
			HR	95 % CI	*p*-value
Site	Colon	923/2847	1.00	-	-
	Rectum	487/1518	0.85	0.74-0.98	0.022
Stage dukes	A	55/933	1.00	-	-
	B	250/1438	2.97	2.21-3.99	0.000
	C	520/1345	7.74	5.75-10.4	0.000
	D	585/649	34.1	25.0-46.5	0.000
Grade	Low	10/114	1.00	-	-
	Intermediate	826/3137	1.22	0.72-2.07	0.453
	High	405/825	2.25	1.32-3.84	0.003
	Unknown	159/289	2.09	1.20-3.64	0.010
Age group	50-59	298/901	1.00	-	-
	60-69	467/1552	1.04	0.89-1.21	0.600
	70-79	645/1942	1.12	0.96-1.29	0.146
Sex	Female	585/1867	1.00	-	-
	Male	825/2498	1.07	0.95-1.20	0.243
Private Insurance	No	686/1985	1.00	-	-
	Yes	724/2380	0.95	0.84-1.06	0.335
Comorbidity	None	941/3068	1.00	-	-
	One (not severe)	248/779	0.90	0.78-1.05	0.194
	Multiple / severe	221/518	1.21	1.02-1.44	0.033
SES quintile	Low	382/1072	1.00	-	-
	Low-mid	280/878	0.94	0.80-1.11	0.443
	Middle	246/791	0.93	0.78-1.10	0.379
	Mid-high	276/843	1.06	0.90-1.25	0.508
	High	226/781	0.75	0.62-0.91	0.004
Residence	Urban	1007/3102	1.00	-	-
	Outer urban	122/429	0.95	0.79-1.16	0.638
	Rural	47/188	0.98	0.82-1.17	0.830
	Remote	93/242	1.12	0.90-1.39	0.324
Surgery^a^	No	230/295	1.00	-	-
	Yes	1180/4070	0.51	0.42-0.62	0.000
Radiotherapy^a^	No	1141/3757	1.00	-	-
	Yes	36/269	1.41	1.18-1.68	0.000
Chemotherapy^a^	No	632/2745	1.00	-	-
	Yes	778/1620	0.87	0.76-1.00	0.047
Diagnosis year	(continuous)	1410/4365	0.95	0.92-0.98	0.002

CRC patients age 50-79 years diagnosed in South Australia 2003–3008

Analysis with multiply imputed stage and grade, where missing, revealed similar results (i.e. identical patterns with regard to significant associations and approximately equivalent point estimates for all HRs)

^a^Treatments received within 12 months of diagnosis

**Fig. 2:**
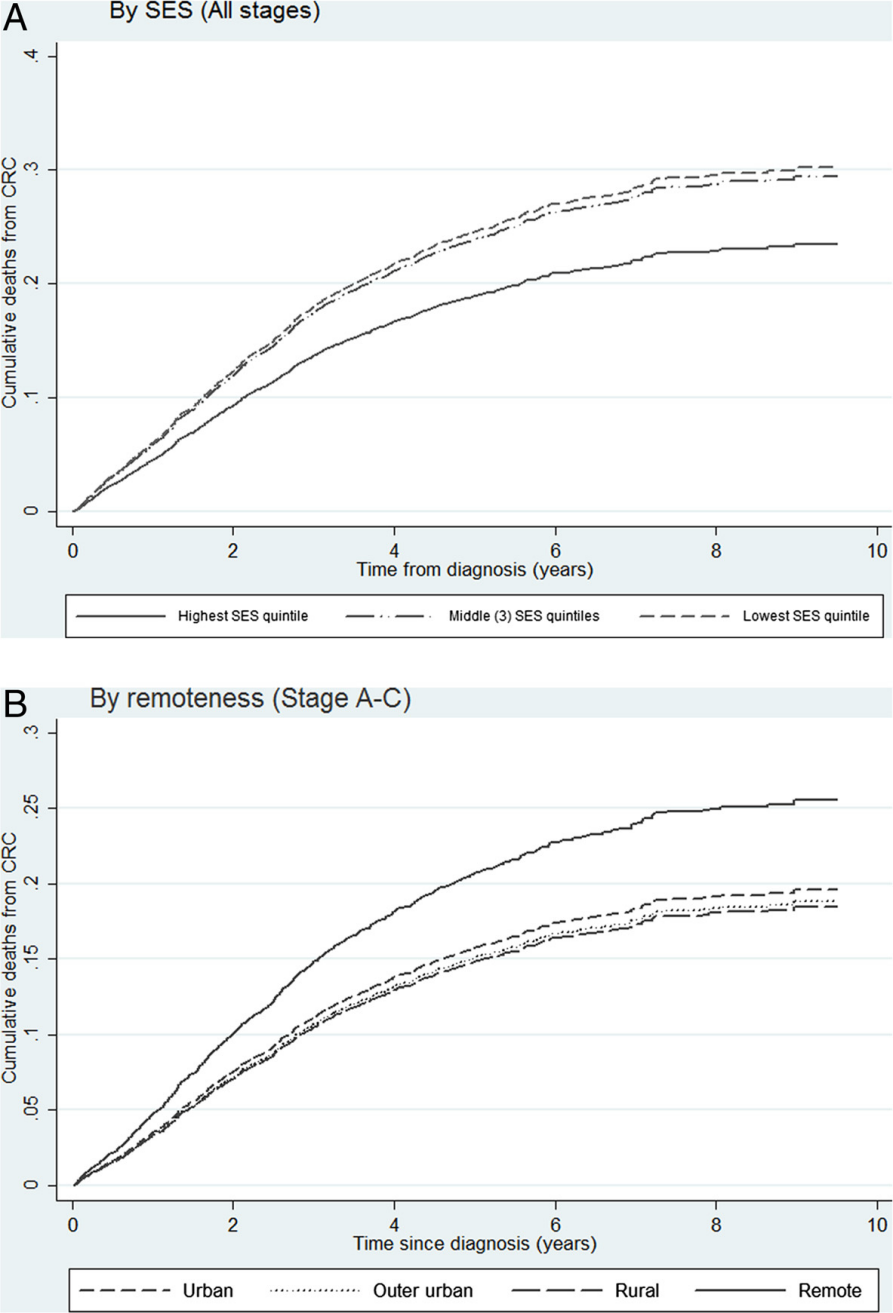
Cumulative CRC mortality by **a** SES quintiles (for all cases 3 middle quintiles collapsed) and **b** remoteness of residence (cases with stages A-C at diagnosis) Fig. 2a and b represent cumulative incidence plots for CRC mortality by based on competing risk regression, for CRC cases diagnosed 2003-2008 among South Australians aged 50-79 yrs. Covariates include age, tumour site, stage, grade, co-morbidity, health insurance status, treatments received in first 12 months, and diagnosis year, categorised as described in methods. SES quintiles low-mid, mid, mid-high were collapsed into one category

Results of stratified analyses, restricted to cases with potentially curable (stages A-C), or metastatic disease (stage D), are shown in Tables 5 and 6. For potentially curable disease, risk of CRC death was higher among residents from remote regions compared with metropolitan residents (HR = 1.35, 95 % CI 1.01-1.80). Regional differences were not evident among patients with stage D CRC at diagnosis. Risk CRC death was lower among those in the highest compared with lowest socioeconomic quintile, both among those with potentially curable CRC (HR = 0.80, 95 % CI 0.63-1.01, non-significant) and for those with metastatic cancer (HR = 0.65, 95 % CI 0.47-0.89). Also private insurance was associated with improved outcomes (HR = 0.80, 95 % CI 0.67-0.96) among patients with metastatic disease at diagnosis but no difference was observed for those with stage A-C CRC. There was no evidence of improvement in survival for metastatic CRC patients over the study period, whereas there was a trend toward improved survival (reduced risk of death) over time for patients with stage A-C CRC (HR = 0.93, 95 % CI 0.90-0.97). Figure 2 shows the cumulative mortality plots for CRC mortality, adjusted for covariates and competing risk of death from other causes by neighbourhood SES, for all cases, and place of residence for stage A-C CRC.

**Table 5:** Multivariate competing risks regression analysis for risk of death from CRC, for stages A-C

Covariates		No. CRC deaths/total	Stages A-C (*n* = 3716)		
			HR	95 % CI	p-value
Site	Colon	523/2409	1.00	-	-
	Rectum	302/1307	0.94	0.78-1.14	0.538
Stage dukes	A	55/933	1.00	-	-
	B	250/1438	2.93	2.18-3.94	0.000
	C	520/1345	6.72	4.92-9.17	0.000
Grade	Low	12/104	1.00	-	-
	Intermediate	512/2779	1.09	0.61-1.95	0.779
	High	262/670	2.13	1.18-3.87	0.013
	Unknown	39/163	1.59	0.82-3.07	0.172
Age group	50-59	172/758	1.00	-	-
	60-69	282/1317	1.08	0.89-1.31	0.433
	70-79	371/1641	1.12	0.93-1.36	0.240
Sex	Female	348/1604	1.00	-	-
	Male	477/2112	1.09	0.94-1.26	0.243
Private Insurance	No	352/1625	1.00	-	-
	Yes	473/2091	1.07	0.92-1.24	0.378
Comorbidity	None	580/2672	1.00	-	-
	One (not severe)	142/653	0.94	0.77-1.13	0.504
	Multiple / severe	103/391	1.29	1.02-1.62	0.032
SES quintile	Low	216/890	1.00	-	-
	Low-mid	164/749	0.98	0.79-1.20	0.819
	Middle	142/678	0.80	0.63-1.00	0.048
	Mid-high	169/730	1.00	0.80-1.24	0.967
	High	134/669	0.80	0.63-1.01	0.057
Residence	Urban	578/2624	1.00	-	-
	Outer urban	74/374	0.96	0.74-1.23	0.724
	Rural	117/515	0.94	0.75-1.17	0.561
	Remote	56/203	1.35	1.01-1.80	0.040
Surgery^a^	No	41/96	1.00	-	-
	Yes	784/3620	0.38	0.26-0.56	0.000
Radiotherapy^a^	No	656/3217	1.00	-	-
	Yes	169/499	1.48	1.16-1.88	0.002
Chemotherapy^a^	No	399/2487	1.00	-	-
	Yes	426/1229	1.11	0.92-1.35	0.284
Diagnosis year	(continuous)	825/3716	0.93	0.90-0.97	0.002

CRC patients age 50-79 years diagnosed in South Australia 2003–2008

Analysis with multiply imputed stage and grade, where missing, revealed similar results (i.e. identical patterns with regard to significant associations and approximately equivalent point estimates for all HRs)

^a^Treatments received within 12 months of diagnosis

**Table 6:** Multivariate competing risks regression analysis for risk of death from CRC, for stage D CRC

Covariates		No. CRC deaths/total	Stage D (n = 649)		
			HR	95 % CI	p-value
Site	Colon	400/438	1.00	-	-
	Rectum	185/211	0.71	0.58-0.87	0.001
Stage dukes	A		-	-	-
	B		-	-	-
	C		-	-	-
Grade	Low	8/10	1.00	-	-
	Intermediate	314/358	1.25	0.53-2.94	0.605
	High	143/155	2.04	0.86-4.83	0.105
	Unknown	120/126	2.26	0.94-5.42	0.067
Age group	50-59	126/143	1.00	-	-
	60-69	185/205	1.15	0.90-1.47	0.254
	70-79	274/301	1.15	0.90-1.46	0.261
Sex	Female	237/263	1.00	-	-
	Male	348/386	1.11	0.92-1.33	0.295
Private Insurance	No	334/360	1.00	-	-
	Yes	251/289	0.80	0.67-0.96	0.016
Comorbidity	None	361/396	1.00	-	-
	One (not severe)	106/126	0.87	0.68-1.11	0.259
	Multiple / severe	118/127	1.13	0.87-1.45	0.364
SES quintile	Low	166/182	1.00	-	-
	Low-mid	116/129	0.85	0.65-1.11	0.231
	Middle	104/113	1.07	0.81-1.41	0.622
	Mid-high	107/113	1.15	0.90-1.48	0.259
	High	92/112	0.65	0.47-0.89	0.007
Residence	Urban	429/478	1.00	-	-
	Outer urban	48/55	1.00	0.74-1.34	0.975
	Rural	71/77	1.05	0.79-1.41	0.724
	Remote	37/39	0.91	0.67-1.23	0.553
Surgery^a^	No	189/199	1.00	-	-
	Yes	396/450	0.58	0.46-0.72	0.000
Radiotherapy^a^	No	485/540	1.00	-	-
	Yes	100/109	1.25	0.98-1.58	0.068
Chemotherapy^a^	No	233/258	1.00	-	-
	Yes	352/391	0.62	0.51-1.04	0.000
Diagnosis year	(continuous)	585/649	0.99	0.94-1.04	0.619

## Discussion

This study examined factors associated with disease-specific survival in a population-wide cohort of CRC patients with access to universal healthcare, with specific focus on identifying socioeconomic and regional disparities. As expected CRC survival was strongly associated with tumour characteristics at diagnosis (i.e. poorer outcomes for more advanced stage and higher grade). However we also found evidence of area level socioeconomic and regional differences in survival for certain subgroups of patients, after accounting for clinical factors including stage. Outcomes were significantly better for those living in the highest compared with lowest SES neighbourhoods, with a more pronounced effect for metastatic disease. We also observed better outcomes among privately insured patients with metastatic disease. Significantly worse outcomes were observed among those living in remote compared with metropolitan regions, though this applied to potentially curable disease only.

While our results suggest that CRC survival differs according to SES, despite provision of universal healthcare services, some observations weaken this conclusion. There was no clear ‘dose response’ with increasing SES. Among stage A-C cases, risk differences for the highest compared with lowest SES areas did not reach statistical significance when restricted to those with stage A-C disease. Furthermore, our measure of SES was based on characteristics of an individual’s neighbourhood, rather than individual measures such as the person’s education, occupation or income level. Thus, there is potential for misclassification of SES at the individual level, which may have led to inaccurate findings.

The existing body of international literature showing socioeconomic inequalities in CRC survival, across numerous countries with differing health care systems [7–12], supports our findings. Many of these studies show that disparities persist after adjustment for differences in stage [20]. Evidence from Australian-based studies is mixed. Hall et al. (Western Australia) [33] and Jorgensen et al. (New South Wales) [34] found no association between SES and CRC outcomes, while Kelsall et al. (Victoria) [35], Morris et al. (Western Australia) [36] and Baade et al. (Queensland) [18] did observed socioeconomic disparities. All used area-level measures of SES compiled by the ABS. Our findings provide further evidence of inequalities in CRC according to area-level deprivation in Australia, despite universal access to health care, with similar effect sizes to previous Australian studies (~25 % difference for highest to lowest SES groups) but slightly lower than international estimates [20].

Various explanations for socioeconomic disparities in CRC survival include differences in screening patterns, distribution of stage of disease, lifestyle factors, co-morbidities, access to health care services and inequalities in treatments. In their review, Aarts et al. note the difficulty in attributing SES differences in survival to any one specific factor [20]. Likewise, Palmer and Schneider highlight socioeconomic disparities across the spectrum of colorectal cancer control including early detection, diagnosis, treatment and outcomes, and call for a multidisciplinary approach to tackling inequalities [21]. Since we found no evidence of differences in stage distribution according to SES, it is unlikely that disparities are due to more advanced disease stage in areas with low SES. Furthermore we adjusted for stage in our regression models. While we also adjusted for co-morbidities using the Charlson co-morbidity index, we were unable to adjust for general lifestyle factors such as obesity, tobacco use, lack of physical activity, due to the lack of individual level data, so cannot rule out the influence of these factors. Our previous research found no overall difference in compliance with treatment guidelines, though radiotherapy for rectal cancer was less likely among more advantaged groups [37]. No differences were observed for surgery or chemotherapy by SES. Differences in treatment (at the broad level) do not explain survival patterns in the current study. Furthermore, inclusion of treatments in regression models did not attenuate the observed associations with survival outcomes. However, other treatment related factors like continuation of chemotherapy, resection of liver metastases and access to follow-up care may vary by SES. Further investigation is warranted to determine the mediating pathways for these disparities.

Our study also found poorer outcomes for those from remote locations for stage A to C disease. Regional disparities were not observed for metastatic (stage D) disease, which is consistent with previous finding from clinical registry data [38]. Some caution in our interpretation is warranted given we did not observed any significant differences in the whole study population. Distance from treatment facilities has previously been shown to impact CRC survival in Australia [17, 18], and internationally [13–16]. Baade et al. [17], found that risk of mortality from rectal cancer increased by 6 % with every 100 km distance from a radiotherapy centre for rectal patients in Queensland (Australia). In a subsequent study the same authors also found that CRC patients residing in remote locations had 14 % lower 5 yr-survival compared with their urban counterparts [18]. Similar to our finding, they noted disparities only in relation to residents from remote locations. They hypothesise that regional differences may be due to patient factors (lifestyle factors and co-morbidities) as well as management patterns, and clinical experience in regional hospitals. We were unable to identify any obvious pathway through which regional disparities in survival could be explained. Poorer survival among patients from remote areas with stage A-C CRC suggest inequalities in access to, or quality of, care rather than regional differences in stage at diagnosis. Findings from our previous patterns of care study (in the same cohort) indicated that rural and remote patients with stage C colon cancer were less likely to receive chemotherapy [37], which may be one reason for poorer outcomes. Differences in follow-up care after initial treatment by place of residence may be another explanation. Again further research to address the causes of poorer survival among potentially curable patients from more remote areas is required.

We also observed differences in survival according to treatment modalities. Patients who underwent surgery or received chemotherapy (for Stage D disease) had better survival while patients who recieved radiotherapy had poorer outcomes than those not receiving these treatments. Reasons for better outcomes with surgery are likely to relate to patients’ fitness for surgery or inoperability of the tumour. Likewise receipt of chemotherapy is less likely among frail or unfit patients, who are also likely to have poorer survival outcomes. Thus the results would be consistent with decisions not to offer chemotherapy when there is likely to be limited effectiveness, high risk of side effects, or both. Poorer cancer specific survival among radiotherapy recipients may be due to radiotherapy being given with palliative rather than curative intent in some cases.

### Strengths and limitations

This study demonstrates the utility of record linkage, using administrative and health surveillance databases, to evaluate disparities in outcomes for cancer patients. Record linkage offers considerable advantages over institutionally-based studies when examining disparities according to socioeconomic status or place of residence [39]. The inclusion of all eligible patients, regardless of whether they attended public or private health care services or both, greatly reduces the potential for selection bias to have influenced our results.

Our findings in relation to SES are based on area-level rather than individual level measures of SES, so may not accurately measure the level of socioeconomic disadvantage for individuals. Ideally, individual measures such as income, education level, housing and car ownership would be preferable, however these data are hard to obtain at a population level. Area level measures of neighbourhood deprivation are commonly used to identify socioeconomic disparities at the population-wide level. IRSAD is a rigorously constructed, composite measure of socioeconomic advantage and disadvantage based on census data for small areas which has been widely used to assess SES disparities in Australia [40]. Previous data linkage studies have been hampered by lack of staging data or incomplete information on treatments, particularly those provided through the private health sector. While we made use of staging data from a previous research project, cancer registries across Australia do not routinely collect stage. High priority needs to be given to developing sustainable systems to collect and record staging information within central cancer registries to enhance their value and usefulness in health outcomes research. Cancer Australia initiatives to pilot the collection of staging information in Australian cancer registries are promising developments [41].

Concerns are often raised about the quality and consistency of administrative data, particularly in relation to treatments and comorbidity [42]. Linkage across multiple data sources including hospital admission records, clinical registries and public and private radiotherapy services enhanced our ability to capture broad level treatment information. Our study would have been further enhanced had we had access to national pharmaceutical prescription data, through linkage with the national database, to better ascertainment of chemotherapy administered in outpatient and community settings [43]. The establishment of data integrating authorities, which is underway throughout Australia, will provide greater access to such data which are crucial for evaluating effectiveness of cancer care [44]. While we acknowledge the limitations of using administrative data sources to determine treatment information, undertaking case note reviews at a population level would be impractical and the cost prohibitive. Linkage derived measures are likely to be sufficient for epidemiological studies, though further research is warranted to valid measures and determine the impact of inaccuracies on population-based research.

## Conclusion

This study points to disparities in CRC survival in South Australia according to socioeconomic characteristics and remoteness of patients’ place of residence, despite universal access to healthcare. The reasons for these disparities remain unclear, but do not appear to be due to differences in stage or other prognostic factors, age, comorbid disease burden or treatment difference at a broad level, though access to chemotherapy may be a contributing factor. Our analyses were unable to account for individual lifestyle risks, treatment differences at a more detailed level, hospital/clinician volume or access to follow-up care, some or all of which may be contributing to these disparities. Further research is required to identify the underlying causes of these disparities which can be addressed to achieve equitable outcomes for all patients irrespective of social position or place of residence.

## Abbreviations

ACPS: Australian clinico-pathologic staging
CCI: Charlson Comorbidity Index
CI: confidence interval
CRC: colorectal cancer
HBCR: hospital-based cancer registry
HR: hazard ratio
IRSAD: Index of Relative Socioeconomic Advantage and Disadvantage
NBCSP: National Breast Cancer Screening Program
OR: odds ratio
SA: South Australia
SACR: South Australian Cancer Registry
SES: socioeconomic status
